# S8-3 How do national sport federations implement health promotion? A case study analysis in France

**DOI:** 10.1093/eurpub/ckad133.040

**Published:** 2023-09-11

**Authors:** Benjamin Tezier, Aurélie Van Hoye, Mathieu Winand, Florence Rostan, Fabienne Lemonnier, Stacey Johnson, Anne Vuillemin

**Affiliations:** Université de Lorraine; Université de Lorraine; Lunex University; Santé publique France; Santé publique France; Université Côte d'Azur; Université Côte d'Azur

## Abstract

**Purpose:**

Sports club’s health promotion (HP) has been investigated, illustrating their need for resources, such as support from national sports federations (NSF), to develop into health-promoting settings. The present study investigates how French NSF promote health.

**Methods:**

A two-step case study design was undertaken. A website analysis of 51 NSF, recognized as health promoting by the Olympic Committee, was executed to examine HP visibility, presence in strategic plans, committees and programs. Based on this search, four NSF were chosen for an in-depth study, including interviews with representatives and a document analysis. Data were analyzed based on the Ottawa Charter strategies for HP.

**Results:**

NSF are committed to HP, but HP is not mentioned directly, not visible, nor properly understood. Rather, a health topic approach is adopted, specifically focusing on strategies to enhance social and physical health. HP implementation lacks coordination, where resource investment is based on the sports ministries focus on specific health topics, in a reactive manner, to implement national policies. NSF’s implementation of HP is aligned with findings from sports clubs, limiting further contributions towards guidelines to support HP in their affiliated clubs.

**Conclusions:**

Future research should investigate determinants of HP implementation among NSF to increase their pro-active approach to promote health. Practical implications include a recognition of HP beyond health topics, an integration of health as a transversal aim in NSF’s policies and programs and a governance system coordinating HP activities.

**Funding:**

The work was supported through a partnership between Santé Publique France, Université Côte d’Azur and the Université de Lorraine.

